# Impact of various media and organic carbon sources on biofuel production potential from *Chlorella* spp.

**DOI:** 10.1007/s13205-016-0434-6

**Published:** 2016-05-31

**Authors:** Amit Kumar Sharma, Pradeepta Kumar Sahoo, Shailey Singhal, Alok Patel

**Affiliations:** 1 Biofuel Research Laboratory, Institute of Alternative Energy Research, University of Petroleum and Energy Studies, Bidholi, Dehradun, Uttrakhand 248007 India; 2 Department of Chemistry, University of Petroleum and Energy Studies, Bidholi, Dehradun, Uttrakhand 248007 India; 3 Department of Biotechnology, Molecular Microbiology Laboratory, IIT Roorkee, Roorkee, 247667 India

**Keywords:** *Chlorella* sp., Culture medium, Organic carbon sources, Lipid production, Biofuel

## Abstract

In this study, five *Chlorella species* (*Chlorella vulgaris*, *Chlorella minutissima*, *Chlorella pyrenoidosa*, *Chlorella* sp.1 and *Chlorella* sp.2) were grown in various nutrient medium including BG-11, BBM, Fog’s medium and M_4_N medium for the evolution of biomass and lipid production potential. Among the tested medium, BG-11 was found most economical and efficient medium for all *Chlorella* species. To see the impact of organic carbon sources on lipid production potential, all microalgae species were also cultured in selected medium (BG-11) with different organic carbon sources like glucose, glycerol, sodium acetate, and sucrose under mixotrophic condition. The results showed that all *Chlorella* species performs better under mixotrophic condition, but *Chlorella vulgaris* achieved maximum lipid productivity (3.5 folds higher) in glycerol supplemented culture medium than control medium among all species.

**Electronic supplementary material:**

The online version of this article (doi:10.1007/s13205-016-0434-6) contains supplementary material, which is available to authorized users.

## Introduction

Current research scenario has been focused on sustainable and renewable biofuels due to depletion of world crude oil reserves and hike in fossil fuels prices (Williams et al. 2009; Sajid et al. 2016). Among various sources of renewable energy, microalgae are getting more attention as liquid (biodiesel and bioethanol) and gaseous (biogas and hydrogen) sources of renewable fuels (Prajapati et al. 2014). Microalgae has various advantages over other sources, such as having high lipid productivity, devoid of any seasonal changes, less water and land requirement and greater photosynthetic efficiency, which make it more sustainable. Besides autotrophic growth, microalgae also grow well on various cheap carbon sources such as wastewater (Prajapati et al. 2013), in hetrotrophic mode.

Cultivation condition of microalgae affects both the biomass and lipid productivity along with cost-effectiveness. The composition of nutrient media (Nitrogen, carbon, phosphorus and trace metals) is one of the most significant factors that affect growth parameter and biochemical composition of microalgae (Lam and Lee 2012; Li et al. 2012; Prathima Devi et al. 2012; Wang et al. 2014; Lin and Wu 2015). The effect of different media composition (Allen medium, Bold’s Basal Medium, BG-11 medium, F–Si medium, SK medium, Walne’s medium) on growth rate and lipid accumulation of *C. pyrenoidosa* was studied by Wang et al. (2014) and found F–Si medium is the best and most economical medium for *C. pyrenoidosa* under outdoor conditions (Wang et al. 2014). Huerlimann et al. cultured *Isochrysis* sp., *Nannochloropsis* sp., *Tetraselmis* sp., and *Rhodomonas* sp. microalgae species in three growth medium (L1, f/2, and K-medium) to study the differences in biomass yield, lipid content and lipid productivity. Hence, it is necessitated to investigate and select suitable medium and nutrient level for the cultivation of microalgae with rapid growth and higher lipid content (Huerlimann et al. 2010). In addition, it was also reported that biomass and lipid production of microalgae is also greatly affected by different carbon sources and concentration (Cheirsilp and Torpee 2012; Lam and Lee 2012; Gim et al. 2014; Choi and Yu 2015). The mixotrophic cultivation, using both organic and inorganic (CO_2_) carbon sources in presence of light, is a good strategy to obtain large biomass and higher lipid content (Gao et al. 2010; Bhatnagar et al. 2011; Choi and Yu 2015; Lin and Wu 2015).

The aim of this study is to select and modify growth medium for enhancing biomass and lipid production of *Chlorella vulgaris*, *Chlorella minutissima*, *Chlorella pyrenoidosa*, *Chlorella* sp. 1 and *Chlorella* sp. 2 towards biodiesel production. First, to identify suitable medium, all *Chlorella* species were grown in BG-11, BBM, Fogg’s medium and M_4_N. Then, to study the effect of different carbon sources (Fructose, Glycerol, Glucose and Sodium acetate) on growth and lipid content, all the five *Chlorella* species were cultivated in BG-11 nutrient medium under mixotrophic condition.

## Material and methodology

### Microalgae strain and pre-cultivation conditions

Pure cultures of *Chlorella vulgaris*, *Chlorella minutissima*, *Chlorella pyrenoidosa* and *Chlorella* sp. *2* were procured from Vivekananda Institute of Algal Technology (VIAT), Chennai (India); Centre for Conservation and Utilization of Blue Green Algae, IARI New Delhi (India), National Collection of Industrial Microorganisms (NCIM), National Chemical Laboratory, Pune (India) and Yogi Vemana University, Vemanapuram, Kadapa, Andhra Pradesh, India, respectively. A microalgal species was also collected from a pond nearby the area of Dehradun, Uttarakhand and purified at agar plates by standard protocol (Guillard 2005). It was identified morphologically as *Chlorella* sp. and is represented here as “*Chlorella* sp. 1*”*, whereas algae procured from Yogi Vemana University, Vemanapuram, Kadapa, Andhra Pradesh, is represented as “*Chlorella* sp. 2” in this study. The stock cultures of all the strains were maintained regularly on agar slants using sterilized BBM medium (with initial pH of 6.8) under laboratory conditions at 24 °C (±1 °C) under (~2500 lx) light intensity and 16/8 light dark cycle. All the strains were transferred from agar slant into a liquid medium and incubated under the same conditions of temperature and light in photobioreactor. Liquid cultures were used as the inoculum for the designed experiments. The morphology of pure strains was regularly examined under an optical microscope and photographed by a Nikon Eclipse Ci-E microscope (serial no. 59E05).

### Screening and identification of efficient growth media for maximum lipid productivity

The growth performance and lipid productivity of microalgae is highly influenced by the composition of medium used for cultivation. Four media that have been used for photoautotrophic culture of *Chlorella* species include Bold’s basal medium (BBM), BG-11, Fog’s medium and M4N.

*Bold’s basal medium* (Ilavarasi et al. 2011); NaNO_3_ 250 mg/L, K_2_HPO_4_ 75 mg/L, MgSO_4_.7H2O 75 mg/L, CaCl_2_.2H_2_O 25 mg/L, KH_2_PO_4_ 175 mg/L, NaCl 25 mg/L^,^ Alkaline EDTA solution 1 mg/L (Alkaline EDTA solution: 5 g Na_2_-EDTA and 3.1 g KOH in 100 ml distilled water), Acidified Iron solution 1 mg/L (Acidified Iron solution FeSO_4_.7H2O .498 g and 0.1 ml H_2_SO_4_ in 100 ml distilled water) Trace metal solution 1 ml/L (Trace metal solution: MnCl_2_.4H_2_O 1.44 g/L, ZnSO_4_.7H_2_O 8.82 g/L, (NH_4_)_6_ Mo_7_O_24_.2H_2_O 0.88 g/L, Co(NO_3_)_2_.6H_2_O 0.49 g/L, CuSO_4_.5 H_2_O 1.57 g/L).

*BG-11 medium* (Ilavarasi et al. 2011); NaNO_3_ 1500 mg/L, K_2_HPO_4_ 40 mg/L^,^ MgSO_4_.7H2O 75 mg/L, CaCl_2_.2H_2_O 36 mg/L, Citric acid 6 mg/L, Trace metal solution 1 ml/L (Trace metal solution: FeC_6_H_5_O_7_.NH_4_OH 6 g/L, Na_2_-EDTA 1 g/L, MnCl_2_.4H_2_O 1.81 g/L, ZnSO_4_.7H_2_O 0.222 g/L, Na_2_ MoO_4_.2H_2_O 0.39 g/L, CuSO_4_.5H_2_O 0.08 mg/L, H_3_BO_3_ 2.86 g/L.

*Fog medium* (Kothari et al. 2012); KNO_3_ 2000 mg/L, K_2_HPO_4_ 200 mg/L, MgSO_4_.7H2O 200 mg/L, CaCl_2_.2H_2_O 100 mg/L, Fe-EDTA solution 5 ml/L (Fe-EDTA solution: 745 mg Na_2_-EDTA and 557 mg FeSO_4_.7H_2_O in 100 ml distilled water), Trace metal solution 1 ml/L (Trace metal solution: H_3_BO_3_ 2.86 g/L, MnCl_2_.4H_2_O 1.81 g/L, ZnSO_4_.7H_2_O 0.222 g/L, Na_2_ MoO_4_-2H_2_O 0.39 g/L, CuSO_4_.5H_2_O 0.08 g/L,).

*M*_*4*_ *N medium* (Yanagi et al. 1995); KNO_3_ 5000 mg/L, K_2_HPO_4_ 1250 mg/L, MgSO_4_.7H_2_O 25000 mg/L, CaCl_2_.2H_2_O 100 mg/L, FeSO_4_.7H_2_O 30 mg/L, Trace metal solution 1 ml/L (Trace metal solution:, MnCl_2_.4H_2_O 1.81 g/L, ZnSO_4_.7H_2_O 0.222 g/L, Na_2_MoO_4_.2H_2_O 0.39 g/L, CuSO_4_.5H_2_O 0.08 g/L, H_3_BO_3_ 2.86 g/L).

All the *Chlorella species* were cultured in 250 ml Erlenmeyer flasks containing 125 ml liquid using above four nutrient media composition under cool florescent light (~2500 lx) at 24 °C (±1 °C) and 16:8 light dark cycle in a photo bioreactor.

### Effect of organic carbon sources on biomass production and lipid accumulation

Ignoring CO_2_ in air, the effect of different organic carbon sources like glucose, glycerol, sodium acetate, and sucrose) was also tested with selected BG-11 growth media. Carbon content of different organic sources was kept same (0.5 g/L) during the experiment. Sterile media was used during whole experiment. The experiments were conducted at room temperature (~22–30 °C) under cool white, fluorescent light for 14 days. 1 l bottle was used as a lab scale photobioreactor. Working volume of photobioreactor was kept 600 ml with 10 % (v/v) inoculum. The photoperiod was set 16:8 light: dark period with fluorescent illumination of ~3000 lx. The culture was aerated (200 ml/min) by aquarium pump to avoid settling of microalgal biomass. The experiment was performed for 14 days at batch scale. Initial pH of medium was adjusted to be 7.0.

### Analytical methods

#### Cultivation parameters

Microalgae growth was determined by measuring the optical density at 680 nm (OD680) using UV–visible spectrophotometer (Thermo Scientific) daily and related to algal biomass (g/L). For biomass estimation 10 ml sample containing algae was filtered through pre-weighted Whatman GF/C glass fiber filter and dried in oven at 60 °C until constant weight. The dry weight of algae is determined by subtracting from final weight of whatman GF/C glass fiber filter with algae to initial weight.

The biomass productivity P (mg/L/day) was calculated by following equation:

1 $$P = \frac{{{\text{W}}_{2} - {\text{W}}_{1} }}{t}$$

where, W_1_ was the initial biomass concentration, W_2_ was the biomass concentration at the last day of cultivation, *t* was the cultivation time.

#### Lipid extraction and measurement

Lipid was extracted by applying Folch extraction method (Folch et al. 1957). According to this method, a weighted amount of biomass (0.250 mg) was dissolved in 5 ml chloroform/methanol (2:1 v/v) and vortex for 30 s. This was followed by agitating the mixture for 15–20 min at room temperature. The mixture was then centrifuged at 8000 rpm for 10 min to separate cell debris from supernatant. This supernatant was washed by 0.9 % NaCl solution and vortex for few seconds. The mixture was centrifuged at 3000 rpm for 5 min. Lower chloroform layer with lipid was removed carefully and collected in 20 ml pre-weighted glass vial. The residue was re-extracted with 2.5 ml chloroform/methanol (1:1 v/v) thrice as stated above. The supernatant was collected in same vial. The solvent was then dried at 65 °C in oven until constant weight of lipid was achieved. The lipid content was calculated gravimetrically. The lipid productivity (mg/L/d) was calculated by the following equation:

2 $${\text{Lipid}}\;{\text{productivity}} = \frac{{{\text{Lipid}}\;{\text{content}}_{t} \times {\text{W}}_{1} - {\text{Lipid}}\;{\text{content}}_{0} \times {\text{W}}_{2} }}{t} \times 1000$$

where Lipid content_t_ was the lipid content at the last day of cultivation and Lipid content_0_ was the initial lipid content in algal cells.

#### Fatty acid profile analysis

Fatty acid extracted by Folch method was converted to their methyl esters by transesterification. Lipid was dissolved in 1 ml of 1 % NaOH in CH_3_OH and heated for 55 °C for 15 min. Then, 2 ml of 5 % methanolic HCl was added and again heated for 15 min at 55 °C. The obtained product mixture was washed with distilled water. Fatty acid methyl esters (FAME) were extracted using 1 ml hexane thrice and evaporated to dryness (Kumari et al. 2011). FAME was re-dissolved in 200 µl hexane and analyzed using a Gas chromatograph (Nucon 5700 series) equipped with Flame ionization detector (FID) using EOX column (serial no 5061; 30 m × 0.25 mm × 0.25 µm). Pure Nitrogen (99.999 %) used as carrier gas with a flow rate of 1 ml/min and pre-column pressure of 49.7 kPa. The initial temperature was set at 160 °C for 2 min, followed by a 4 °C/min ramp up to 240 °C and maintained for 50 min. The injector and FID detector temperature was set at 240 and 220 °C, respectively. FAME peaks were identified by the comparison of their retention time with authentic standard by GC and quantified by normalization. Methylated heptadecanoic acid was used as an internal standard.

#### Prediction of fuel properties

Fuel properties of biodiesel obtained from *Chlorella* spp. were examined with the help of following equations (Sinha et al. 2016)

3 $${\mathbf{SV}} = \, \varSigma \, \left( { 5 60 \, \times N} \right) /M_{\text{W}}$$

4 $${\mathbf{IV}} = \, \varSigma \, \left( { 2 5 4 { } \times N \times D} \right) /M_{\text{W}}$$

5 $${\mathbf{CN}} = \, \left( { 4 6. 3 { } + { 5458 }/{\text{SV}}} \right) \, - \, \left( {0. 2 2 5 { } \times {\text{IV}}} \right)$$

6 $${\mathbf{DU}} = \, \left( {{\text{MUFA}},{\text{ wt }}\% } \right) \, + \, \left( { 2 { } \times {\text{ PUFA}},{\text{ wt }}\% } \right)$$

$$\begin{aligned} {\mathbf{LCSF}} = \, \left( {0. 1 { } \times {\text{ C 16}}: \, 0} \right) \, + \, \left( {0. 5 { } \times {\text{ C18}}: \, 0} \right) \, + \, \left( { 1 { } \times {\text{ C2}}0: \, 0} \right) \, + \, \left( { 2 { } \times {\text{ C24}}: \, 0} \right) \hfill \\ \end{aligned}$$

7 $$\begin{aligned} {\mathbf{CFPP}} = \, \left( { 3. 1 4 1 7 { } \times {\text{ LCSF}}} \right) - 1 6. 4 7 7\hfill \\ \end{aligned}$$

8 $${\mathbf{OS}} = 1 1 7: 9 2 9 5/{\text{X }} - 2: 5 90 5 { }\left( {{\text{where }}0 < {\text{X}} > 100} \right)$$

9 $${\mathbf{CP}} = \, (0. 5 2 6 { } \times {\text{ C 16 }}{-}{ 4}. 9 9 2)$$

10 $${\mathbf{PP}} = \, \left( {0. 5 7 1 { } \times {\text{ C 16}} - 1 2. 2 40} \right)$$

$${\mathbf{HHV}}\left( {{\mathbf{MJ}}/{\mathbf{kg}}} \right) = { 49}: 4 3 { } - \, 0:0 4 1 { } \times \, \left( {\text{SV}} \right) - 0:0 1 5\left( {\text{IV}} \right)$$

where *N* is the percentage of each fatty acid, *M*_W_ is the molecular mass of fatty acid, *M*_*i*_ is the molecular mass of *i*th fatty acid, *D* is the number of double bonds, MUFA is monounsaturated fatty acids, PUFA is polyunsaturated fatty acid, CFPP is cold filter pluging point, OS oxidation stability, CP cloud point, PP pour point, *X* is the percentage of linoleic and linolenic acids (wt %) and HHV is the calorific value of FAME in MJ/Kg.

### Statistics

The data were recorded in triplicates and represented as mean of three experiments with ±standard deviation.

## Result and discussion

### Characterization of microalgae strains

Five strains of fresh water microalgae (*Chlorella vulgaris*, *Chlorella minutissima*, *Chlorella pyrenoidosa, Chlorella* sp. 1 and *Chlorella* sp. 2) selected for the laboratory experiments belong to the division of Chlorophyta and the class of Chlorophyceae. The pictures of all the five green microalgae were taken by an optical microscope (S1). One microalgae species was isolated from Dehradun region and identified morphologically. Characteristics and Morphological features of the isolated microalgae have its close similarity with genus *Chlorella vulgaris, Chlorella minutissima, Chlorella pyrenoidosa,* and *chlorella* sp. 2 and represented here as *Chlorella* sp. 1. The individual cells of the colonies were in the range of 2–10 µm. Microalgal cells were green color, unicellular, and spherical in shape.

### Screening and identification of efficient growth media for maximum lipid productivity

**Fig. 1:**
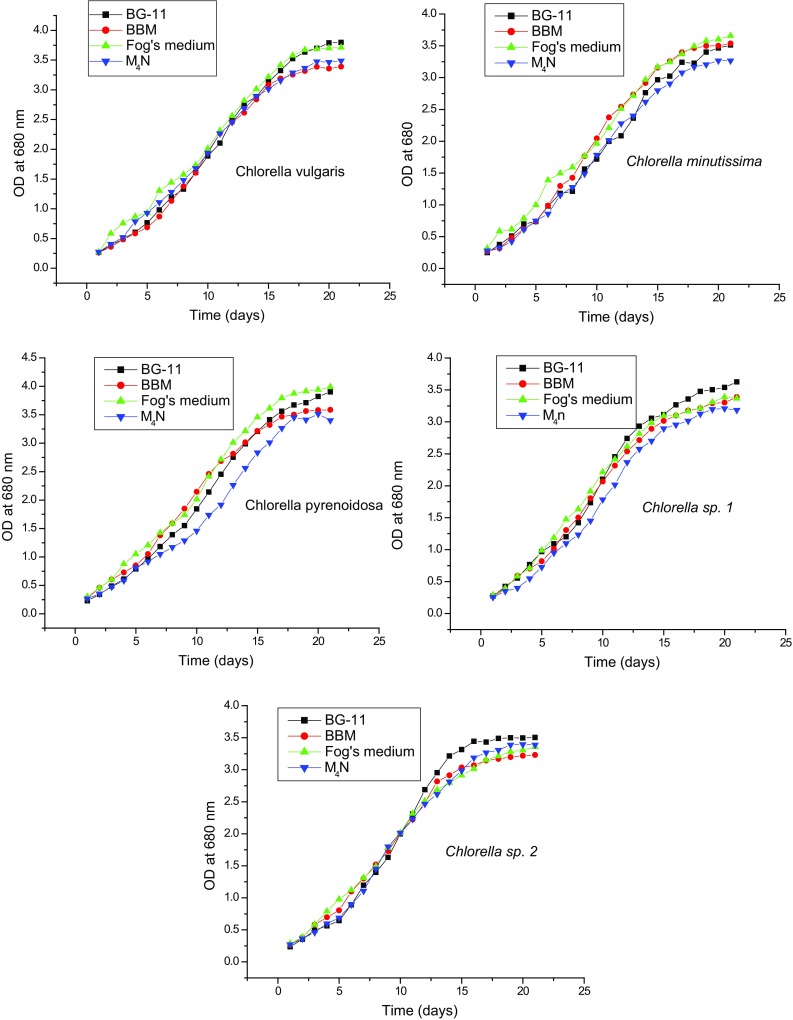
OD of *Chlorella vulgaris*, *Chlorella minutissima*, *Chlorella pyrenoidosa*, *Chlorella* sp. 1 and *Chlorella* sp. 2 biomass in different nutrient media

**Fig. 2:**
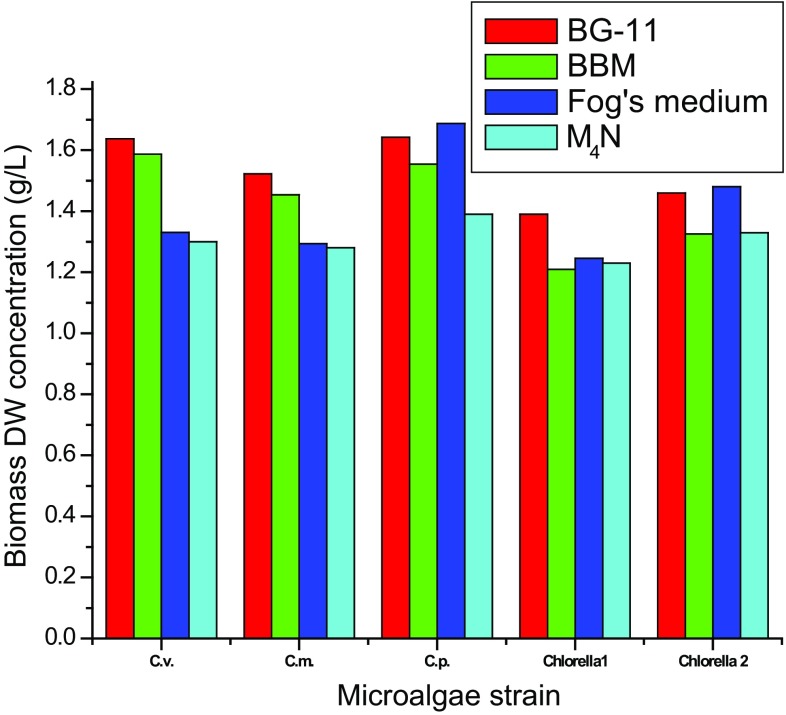
Comparison of biomass DW of five microalgal strains in four culture media. Cv, *Chlorella vulgaris*; Cm, *Chlorella minutissima*; Cp, *Chlorella pyrenoidosa*; Chlorella 1, *Chlorella* sp. 1 and Chlorella 2, *Chlorella* sp. 2

All the microalgae species (*Chlorella vulgaris*, *Chlorella minutissima*, *Chlorella pyrenoidosa*, *Chlorella* sp. 1, and *Chlorella* sp. 2) were cultured in four media i.e. BG-11, BBM, Fog’s and M_4_N medium in 250 ml Erlenmeyer flasks (conical flask) each containing 125 ml media. The results revealed that all the microalgal species had approximately 2 days lag period and reached the exponential phase within 4–6 days in all the media. Microalgae cells achieved stationary phase within 15 days and after that cells growth was very slow as shown in Fig. 1. As each microalgal species shows different biochemical composition (e.g., protein, amino acids, carbohydrate, lipid, fatty acids, chlorophyll and carotenoids) in different media which is reflected by different optical density (OD) of samples. Hence, biomass dry weight (DW) does not correlate well with OD for different culture media or strains. To avoid this error, actual biomass concentration dry weight (DW) was used to study microalgae growth in different media. DW biomass concentration of all the five microalgae species at the stationary phase are shown Fig. 2. It was examined that highest biomass concentration of *Chlorella vulgaris* was in BG-11 (1.64 ± 0.07 g/L), followed by BBM (1.58 ± 0.05 g/L), Fog’s medium (1.33 ± 0.03 g/L) and M_4_N (1.30 ± 0.04 g/L). Similarly, *Chlorella minutissima* (1.52 ± 0.03 g/L) and *Chlorella* sp. 1 (1.39 ± 0.05 g/L) also showed maximum biomass concentration in BG-11. But, in case of *Chlorella pyrenoidosa* and *Chlorella* sp. 2, biomass concentration was maximum (1.69 ± 0.02 and 1.48 ± 0.06 g/L, respectively) in Fog’s medium. This can be explained by the fact that higher nitrogen concentration is favorable for increasing biomass growth (Li et al. 2012). However, M_4_N (having maximum nitrogen concentration) showed poor biomass concentration which is due to deleterious effect of nitrogen at higher concentrations (Li et al. 2012).

**Fig. 3:**
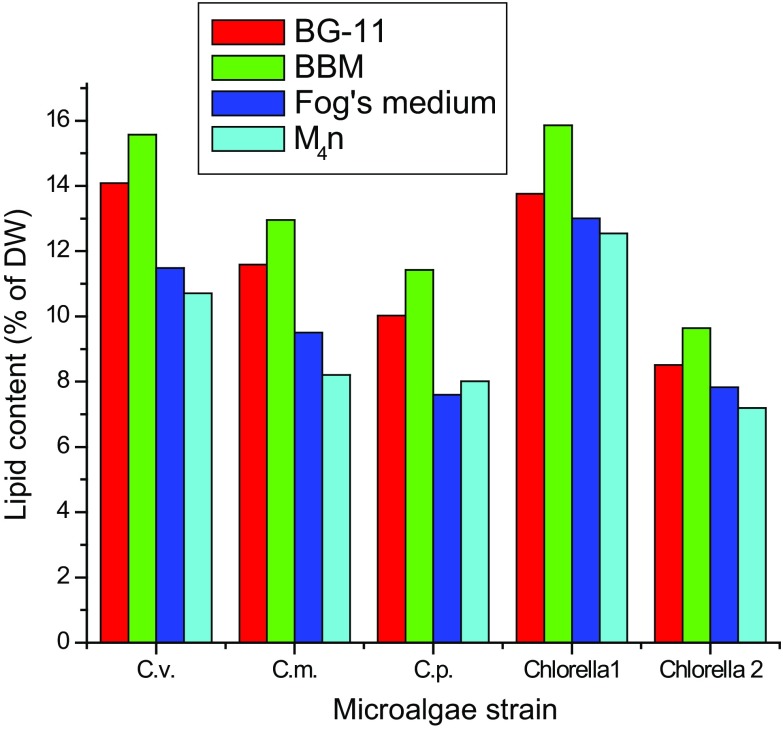
Comparison of lipid content of five microalgal strains in four culture media. Cv, *Chlorella vulgaris*; Cm, *Chlorella minutissima*; Cp, *Chlorella pyrenoidosa*; Chlorella 1, *Chlorella* sp. 1 and Chlorella 2, *Chlorella* sp. 2

**Fig. 4:**
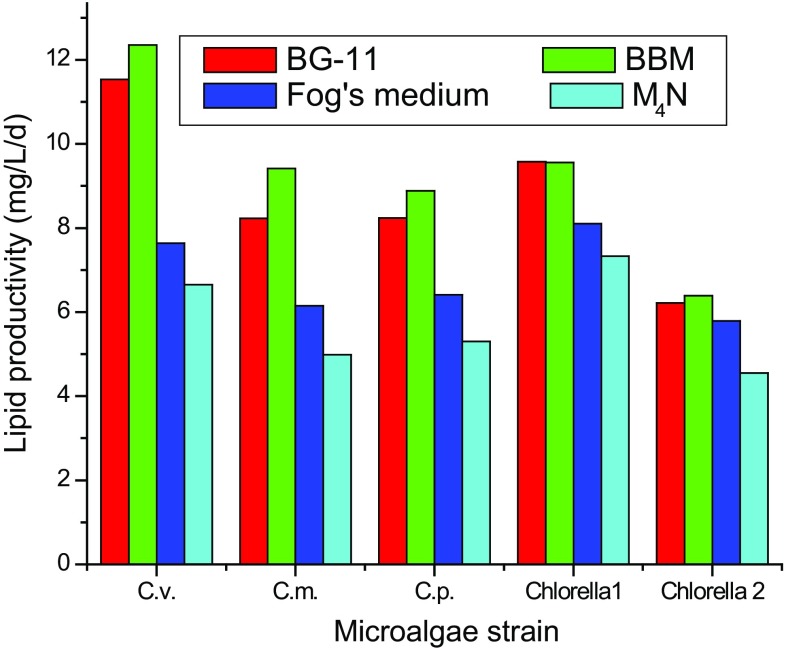
Comparison of lipid productivity of five microalgal strains in four culture media. Cv, *Chlorella vulgaris*; Cm, *Chlorella minutissima*; Cp, *Chlorella pyrenoidosa*; Chlorella 1, *Chlorella* sp. 1 and Chlorella 2, *Chlorella* sp. 2

Lipid content was reported as a percentage of lipids to biomass DW. Similar to biomass yield, significant variances in lipid content were found across strains and culture media. Lipid contents and productivity of all the five strains in different culture media are shown in Figs. 3 and 4, respectively. Maximum lipid content and productivity was found in BBM media for all the *Chlorella* *species*. The highest lipid content of 15.87 ± 0.77 % was found for *Chlorella* sp. 1 cultivated in BBM, followed by *Chlorella vulgaris* (15.57 ± 1.44 %), *Chlorella minutissima* (12.95 ± 0.53 %), *Chlorella pyrenoidosa* (11.43 ± 1.26 %) and *Chlorella* sp. 2 (9.64 ± 0.86 %) in BBM medium. Minimum lipid content was observed in M_4_N growth medium. Furthermore, all microalgae species showed highest lipid productivity in BBM growth media and lowest in M_4_N medium. Among all the tested species, maximum lipid productivity was observed for *Chlorella vulgaris* (12.35 ± 0.37 mg/L/d), followed by *Chlorella* sp. 1 (9.56 ± 0.43 mg/L/d), *Chlorella minutissima* (9.41 ± 0.67 mg/L/d), *Chlorella pyrenoidosa* (8.88 ± 0.41 mg/L/d) and *Chlorella* sp. 2 (6.39 ± 0.35 mg/L/d). These data are supported by the fact that nitrogen starvation condition results in more lipid accumulation (Converti et al. 2009; Chen et al. 2011; Feng et al. 2011; Kumari et al. 2011; Li et al. 2012). The selection of culture medium depends upon various factors viz. the target product, growth rate, and medium cost. However, nitrogen is the key factor in growth medium and also a limiting nutrient affecting the biomass growth and lipid productivity of various microalgae (Griffiths and Harrison 2009). Culture media comparison shows that on an average BBM gave highest lipid content and productivity, followed by BG-11, whereas Fog’s medium and M_4_N had the lowest. Generally, lipid content of microalgae increases when microalgae are subjected to unfavorable culture conditions, such as nutrient starvation (Converti et al. 2009; Chen et al. 2011; Feng et al. 2011). According to Li et al. (2012), the lipid yield of microalgae species can be improved by at least 300 % under nitrogen limited conditions. Since, lipid production is the main aim of microalgae cultivation in this study, BBM and BG-11 are the preferred growth media. BBM had higher lipid content and productivity due to relatively lower nitrogen and phosphate concentrations, whereas, BG-11 medium being nitrogen rich medium has good potential to increase lipid productivity by applying nitrogen deficient condition (Li et al. 2012). Furthermore, total cost of chemicals to prepare 1 l medium was found to be Rs. 0.784 and Rs. 0.472 for BG-11 and BBM growth media, respectively. When applying nitrogen deficient medium (suppose half concentration of nitrogen in case of BG-11) to culture algae, the cost of BG-11 preparation will be reduced up to Rs. 0.460. While BBM is already nitrogen deficient medium and further decrease in nitrogen concentration will results in reduction of total lipid productivity. Therefore, on the basis of potential of growth medium for enhancing lipid productivity and cost reduction, BG-11 had been considered as best medium in this study and used as culture medium for further study.

### Effect of carbon sources on microalgae growth and lipid accumulation

In this study, all the *Chlorella species* were grown in optimized BG-11 medium (optimized in previous research paper, Sharma et al. 2016) with different organic carbon sources at room temperature and 16:8 light:dark cycle. The supplemented organic carbon sources were glucose (7.22 mM), glycerol (14.13 mM), sodium acetate (21.09 mM) and sucrose (3.51 mM) with fixed amount of carbon content (0.5 g/L).

**Fig. 5:**
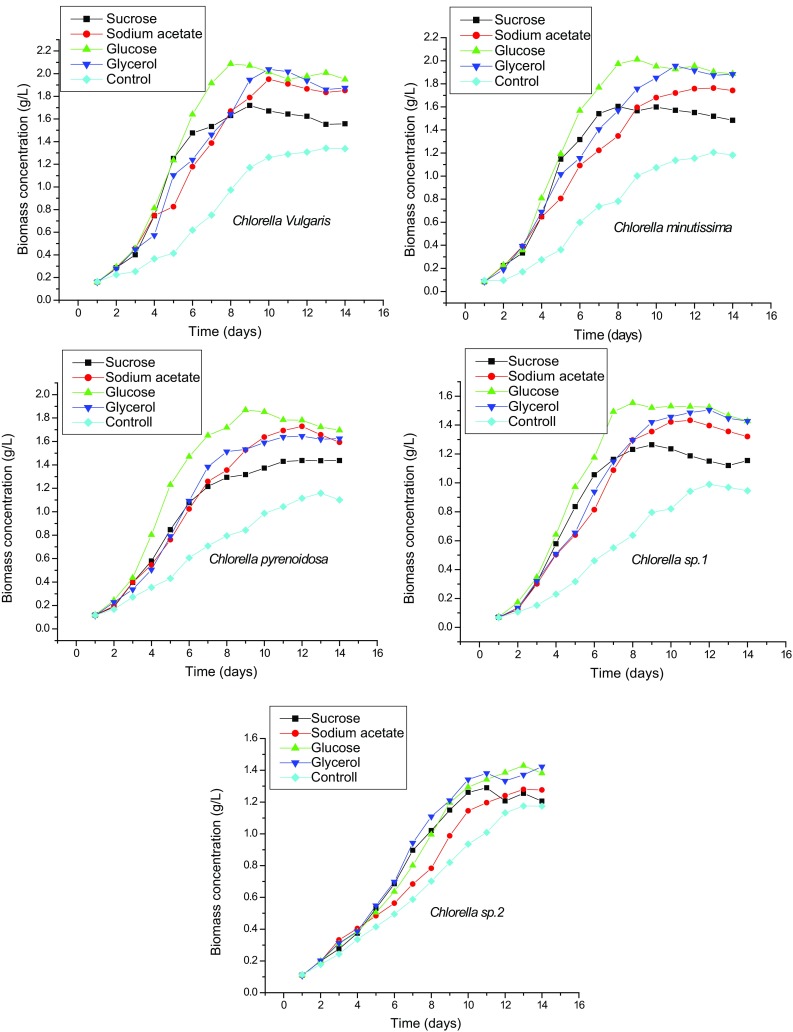
Biomass growths of *Chlorella vulgaris*, *Chlorella minutissima*, *Chlorella pyrenoidosa*, *Chlorella* sp. 1 and *Chlorella* sp. 2 under different carbon sources

**Fig. 6:**
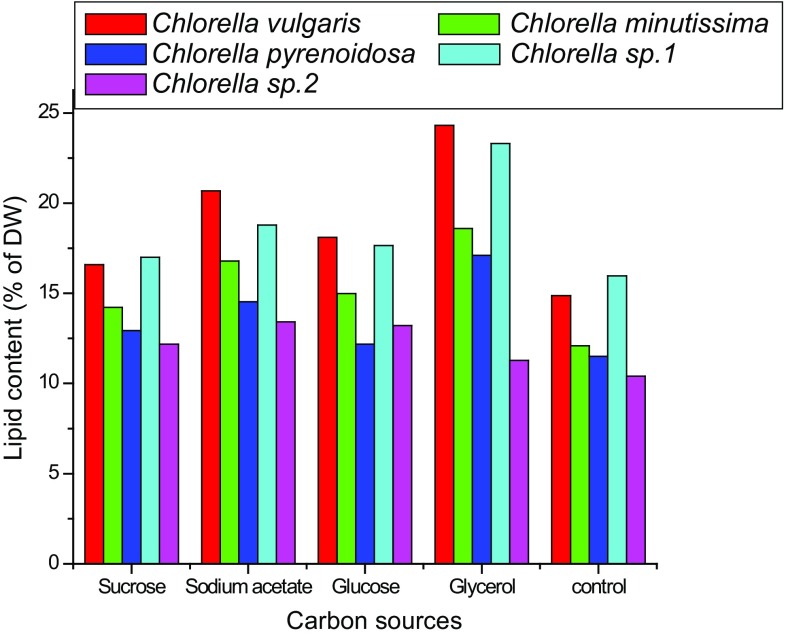
Lipid contents of *Chlorella vulgaris, Chlorella minutissima, Chlorella pyrenoidosa*, *Chlorella* sp. 1 and *Chlorella* sp. 2 under different carbon sources

**Fig. 7:**
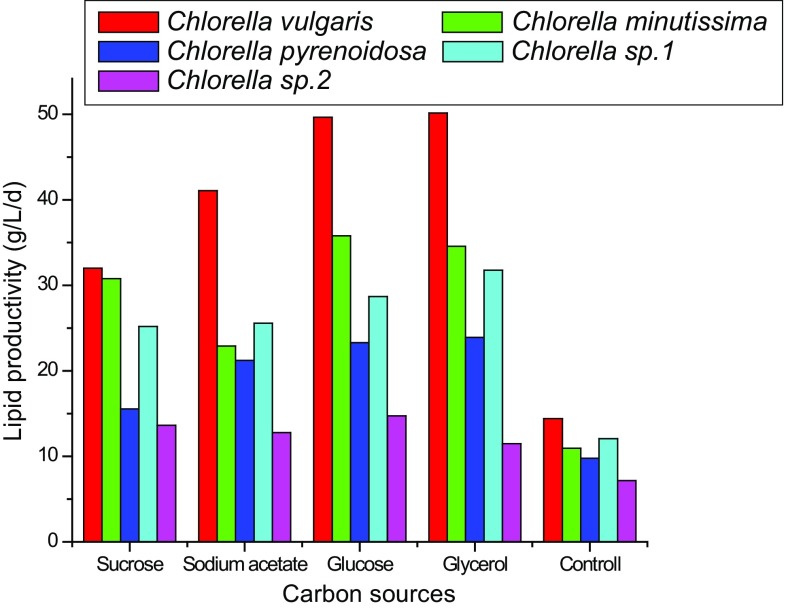
Lipid productivities of *Chlorella vulgaris*, *Chlorella minutissima*, *Chlorella pyrenoidosa*, *Chlorella* sp. 1 and *Chlorella* sp. 2 under different carbon sources

Among the tested organic carbon sources, glucose was found the best carbon source for mixotrophic growth of all the *Chlorella* strains, followed by glycerol, sodium acetate and sucrose. The study showed that addition of glucose results in maximum biomass production of 2.08, 2.04, 1.84, 1.53 and 1.43 g/L for *Chlorella vulgaris*, *Chlorella minutissima*, *Chlorella pyrenoidosa*, *Chlorella* sp. 1 and *Chlorella* sp. 2 at stationary phase (Fig. 5). However, all the microalgae species achieved maximum biomass concentration within 8–11 days in the culture medium supplemented with glucose and the average highest biomass productivity were found 274.46 mg/L/day for *Chlorella vulgaris*, followed by 239.15 mg/L/day for *Chlorella minutissima*, 191.28 mg/L/day for *Chlorella pyrenoidosa*, 162.52 mg/L/day for *Chlorella* sp. 1 and 111.36 mg/L/day for *Chlorella* sp. 1, respectively. Biomass concentration of all the microalgal stains was poor in sucrose culture medium at stationary phase. These results are in agreement with previous report that glucose is an efficient trigger to increase biomass productivity of the microalgae (Perez-Garcia et al. 2011a; Cheirsilp and Torpee 2012; Sun et al. 2014). This can be explained by the fact that glucose is the raw material for photosynthesis and under mixotrophic growth, it can be utilized in the presence of light for energy metabolism for ATP and NAD(P)H production, and, therefore, biomass growth can be accelerated (Yang et al. 2000). On the other hand, lipid contents of four *Chlorella* strains (*Chlorella vulgaris*, *Chlorella minutissima*, *Chlorella pyrenoidosa*, *Chlorella* sp. 1) were significantly higher in culture medium supplemented with glycerol as carbon source and followed by sodium acetate, glucose, and sucrose organic carbon sources, while *Chlorella* sp. 2 showed maximum lipid content in sodium acetate culture medium (Fig. 6). Maximum lipid production was 24.32 % for *Chlorella vulgaris,* 18.59 % for *Chlorella minutissima*, 17.10 % for *Chlorella pyrenoidosa* and 23.31 % for *Chlorella* sp. 1 in glycerol culture medium while 13.42 % *for Chlorella* sp. 2 *in sodium acetate.* Lipid production was found to be lowest in case of sucrose carbon source for all the tested microalgal strains except *Chlorella* sp. 2 which showed exceptional behavior and minimum lipid content of this species was in glycerol. Maximum lipid yield observed for the tested microalgal strains was 490.74 mg/L for *Chlorella vulgaris*, 369.13 mg/L for *Chlorella minutissima*, 282.59 mg/L for *Chlorella pyrenoidosa*, and 325.4 mg/L for *Chlorella* sp. 1 in glycerol culture medium which was more than twofolds higher in comparison to control culture (phototrophic culture) for all these species. On the other hand, *Chlorella* sp. 2 had shown highest lipid yield (188.58 mg/L) in glucose. This can be explained by the fact that glucose is a simple hexose monosaccharide, which is first catabolized into glucose-6-phosphate (important intermediate product for various metabolic precursors) and subsequently to pyruvate through anaerobic glycolysis process, then it enters into tricarboxylic acid cycle (TCA cycle) followed by mitochondrial oxidative phosphorylation for ATPs production (Lu et al. 2011). On the other hand, glycerol enters the cells by simple diffusion without any extra energy, does not behave as a source of carbons for biosynthesis (Perez-Garcia et al. 2011b). The pentose phosphate pathway (PPP) is inhibited during glycerol assimilation (Chen et al. 2011). More glycerol would be used in the Embden–Meyerh of pathway (EMP), and then sacrifice to generate lipids. Furthermore, glycerol is a substrate for triacylglycerol (TAG) synthesis. Overall, it was found that different biomass and lipid productions of all the five microalgal species were due to their different metabolic pathways of carbon and energy sources. Lipid productivity of *Chlorella vulgaris* (50.17 mg/L/d), *Chlorella pyrenoidosa* (23.91 mg/L/d) and *Chlorella* sp. 1 (28.69 mg/L/d) was observed best in glycerol medium, whereas *Chlorella minutissima* (35.8 mg/L/d) and *Chlorella* sp. 2 (14.73 mg/L/d) showed maximum lipid productivity in glucose supplemented culture medium (Fig. 7). But higher cost of glucose is a major hurdle to microalgal biodiesel production economics. Additionally, glycerol is much cheaper than glucose and also a byproduct of biodiesel production. Therefore, utilizing glycerol for microalgae cultivation may be a good approach for recycling it and reduction of biodiesel production cost. Hence, glycerol is more promising candidate to make algae biodiesel economically viable.

### Predicted fuel properties of biodiesel extracted from *Chlorella* species

Fuel properties of biodiesel are highly influenced by fatty acid composition of algae. Fatty acid composition of *Chlorella vulgaris*, *Chlorella minutissima*, *Chlorella pyrenoidosa* *Chlorella* sp. 1 and *Chlorella* sp. 2 is shown in Table 1. Results showed that *Chlorella pyrenoidosa* contains higher saturated FAME (45.07 %) than *Chlorella vulgaris* (31.73 %), *Chlorella minutissima* (35.14 %), *Chlorella* sp. 1 (33.14 %) and *Chlorella* sp. 2 (44.94 %), whereas mono unsaturated fatty acid were found maximum for *Chlorella minutissima* (38.01 %), followed by *Chlorella* sp. 2 (34.53 %), *Chlorella pyrenoidosa* (33.42 %), *Chlorella vulgaris* (21.76 %) and *Chlorella* sp. 2 (17.34 %). Further analysis showed that polyunsaturated fatty acid were 36.14, 25.33, 19.87, 32.36 and 36.19 % in *Chlorella vulgaris*, *Chlorella minutissima,* *Chlorella pyrenoidosa,* *Chlorella* sp. 1 and *Chlorella* sp. 2, respectively. Previous research showed that ideal biodiesel would have low level of polyunsaturated and saturated fatty acids to support the favorable oxidative stability and cold flow problem (Knothe 2009; Hoekman et al. 2012). Therefore, presence of monounsaturated fatty acids, such as palmitoleic acid (16:1) and oleic acid (18:1), play a big role for giving the best compromise between oxidative stability and cold flow (Knothe 2009; Hoekman et al. 2012). In this study, monounsaturated fatty acids were analyzed from 17.34 to 38.01 % for the tested *Chlorella* strains. Among the tested species, *Chlorella minutissima* showed maximum monounsaturated fatty acids (38.01 %) and *Chlorella* sp. 2 showed minimum monounsaturated fatty acids (17.34 %).

**Table 1:** Fatty acid composition of *Chlorella vulgaris, Chlorella minutissima, Chlorella pyrenoidosa, Chlorella* sp. 1 and *Chlorella* sp. 2

Fatty acid composition (%)	*Chlorella vulgaris*	*Chlorella minutissima*	*Chlorella pyrenoidosa*	*Chlorella* sp. 1	*Chlorella* sp. 2
C 8:0	0.1746	0.5943	nd	0.9883	0.4798
C 10:0	0.5171	2.8572	0.2105	1.8319	0.7548
C 12:0	nd	2.1761	1.8167	1.2387	2.6925
C 14:0	3.3011	2.8349	7.2533	2.3923	0.4662
C 16:0	21.5492	19.9060	28.6448	22.0379	36.1705
C 16:1	0.1747	9.4912	nd	4.9447	1.5880
C 18:0	5.0270	4.6787	7.1502	3.7954	5.757
C 18:1	21.5925	28.5275	33.4208	29.5875	15.7582
C 18:2	31.5895	17.8802	14.4817	18.1586	30.6374
C 18:3	4.5529	7.4552	5.3940	14.2094	5.5537
C 20:0	0.4816	0.5592	nd	0.7173	0.6234
C 22:0	0.6832	1.5337	nd	0.1479	nd
Saturated fatty acids	31.7338	35.1401	45.0755	33.1497	44.9442
Mono unsaturated fatty acids	21.7672	38.0187	33.4208	34.5322	17.3462
poly unsaturated fatty acids	36.1424	25.3354	19.8757	32.368	36.1911

It was reported that biodiesel composition enriched with C16–C18 fatty acids have good fuel properties (Knothe 2009). As shown in Table 1, fatty acid compositions of all *Chlorella* strains have major constituent of palmitic (16:0), stearic (18:0), oleic (18:1), linoleic (18:2), and linolenic (18:3) acids. The presence of more polyunsaturated FAME results in lower oxidation stability of biodiesel. This can be explained by the fact that polyunsaturated FAME contains reactive sites which are susceptible for attack of free radicals during oxidation reaction. According to European standards EN14214, for an ideal biodiesel the percentage of linolenic acid (C18:3) and polyunsaturated FA (>4 double bond) should not increase 12 and 1 %, respectively (Knothe 2009). Maximum linolenic acid (C18:3) was found in *Chlorella* sp. 2. (14.20 %) while minimum was for *Chlorella vulgaris* (4.55 %). Furthermore, polyunsaturated FA (>4 double bond) was absent in all the *Chlorella* strains.

Table 2 shows predicted fuel properties of *Chlorella vulgaris*, *Chlorella minutissima* *Chlorella pyrenoidosa Chlorella* sp. 1 and *Chlorella* sp. 2. Iodine value is the measurement of total unsaturation in biodiesel. Higher Iodine Value of the biodiesel may result in the polymerization of glycerides and deposition of lubricant in the engine (Francisco et al. 2010). According to European standards EN 14214, iodine value of biodiesel should not be more than 120 g I2/100 g. In this study, maximum iodine value was observed for *Chlorella* sp. 1 (99.92) while *Chlorella pyrenoidosa* showed minimum iodine value (68.90). Cetane number indicates combustion behavior of fuel in engine. The higher cetane value results in better combustion of fuel, lower NOx emission, less occurrence of knocking and easier start- up of engine. According to ASTMD6751, European (EN 14214) and Australian standard and National Petroleum Agency (ANP255) standard the minimum cetane value should be 47, 51 and 45, respectively. In this study the cetane value of *Chlorella vulgaris*, *Chlorella minutissima*, *Chlorella pyrenoidosa, Chlorella* sp. 1 and *Chlorella* sp. 2 is found 57.10, 54.42, 58.47, 50.88 and 54.68 which satisfied the above standards. Oxidation stability of biodiesel depends of presence of polyunsaturated FAME in biodiesel. This can be explained by the fact that polyunsaturated FAME contains reactive sites which are susceptible for attack of free radicals during oxidation reaction. According to EN 14214, the oxidative stability should be above 6 h. In this case, oxidation stability of five strains recorded 5.8–8.5 h. Cold fuel properties, such as cold filter plugging point, cloud point and pour point, are the most important properties for low temperatures applications. CP is the temperature at which crystallization begins and PP at which fuel no longer pours. These properties are highly affected by the presence of saturated FAME in biodiesel. Higher value of saturated FAME will result in poor cold properties. ASTM D6751 specifies CP range −3 to 12 °C and PP −15 to 20 °C. Five *Chlorella* strains examined in this study showed CP ranges from 6.43 to 14.86 °C and PP from 0.16 to 9.32 °C. Calorific value is the indication of energy produced by the combustion of fuel and it was found to be maximum (40.78 MJ/Kg) for *Chlorella vulgaris* and minimum for followed by *Chlorella sp1* (39.66 MJ/Kg).

**Table 2:** Fuel properties of biodiesel produced from *Chlorella vulgaris, Chlorella minutissima, Chlorella pyrenoidosa, Chlorella* sp. 1 and *Chlorella* sp. 2

Fatty acid composition (%)	*Chlorella vulgaris*	*Chlorella minutissima*	*Chlorella pyrenoidosa*	*Chlorella* sp. 1	*Chlorella* sp. 2
Saponification value (mg KOH g^−^1 oil)	178.68	199.94	197.21	201.63	198.48
Iodine value (g I_2_ 100 g^−1^ oil)	87.73	85.22	68.90	99.92	84.95
Cetane number	57.10	54.42	58.47	50.88	54.68
DU (%)	94.05	88.68	73.17	99.26	89.72
LCSF (°C)	5.15	4.88	6.43	4.81	7.11
CFPP (°C)	−0.29	−1.11	3.75	−1.33	5.88
Oxidation stability (h)	5.8	7.2	8.5	6.2	5.8
Cloud point (°C)	6.43	10.47	10.07	9.20	14.86
Pour point (°C)	0.16	4.54	4.11	3.16	9.32
Higher heating value (MJ/kg)	40.78	39.95	40.31	39.66	40.01

## Conclusion

This study investigates the effect of various growth media and organic carbon sources on biomass and lipid production potential of five *Chlorella* species (*Chlorella vulgaris*, *Chlorella minutissima*, *Chlorella pyrenoidosa*, *Chlorella* sp. 1 and *Chlorella* sp. 2). The results revealed that all the species showed good potential for lipid production in BG-11 supplemented with glycerol in comparison to controlled medium. However, maximum lipid yield was observed for *Chlorella vulgaris* (490.74 ± 12.30 mg/L) among all the tested species. On the basis of lipid productivity, fatty acid composition and fuel properties, it is observed that *Chlorella vulgaris* showed good potential in BG-11 growth media supplemented with glycerol under mixotrophic condition for biodiesel production, but it further needs research for the optimization of C:N:P ratio for large-scale biodiesel production.

## Electronic supplementary material

Below is the link to the electronic supplementary material.

Supplementary material 1 (DOCX 453 kb)
