# Supplementary figures and images for: Impact of various media and organic carbon sources on biofuel production potential from Chlorella spp
Source: 3 Biotech. 2016 May 31;6(2):116. doi: 10.1007/s13205-016-0434-6 (PMC4909020; doi:10.1007/s13205-016-0434-6)

Fig.1


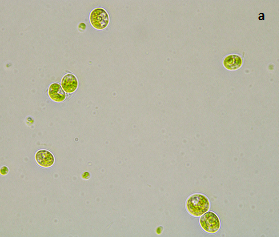

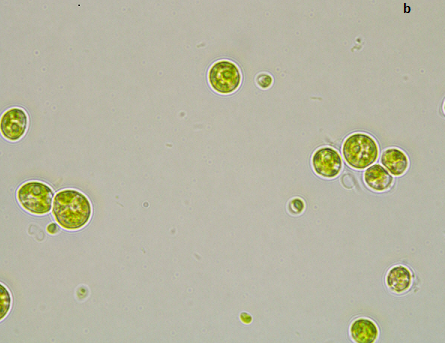


b

a


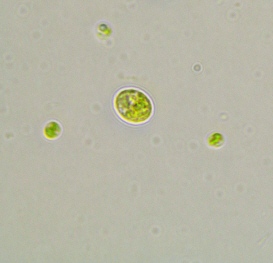

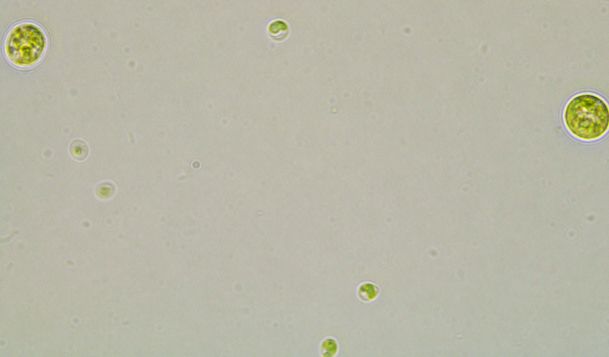


d

c


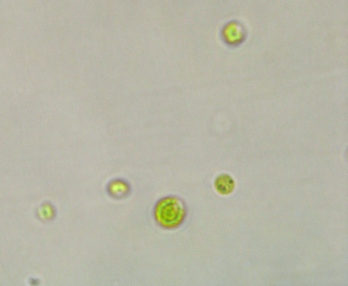


e

Supplement: Supplementary file 1 — Supplementary material 1 (DOCX 453 kb) [file 13205_2016_434_MOESM1_ESM.docx]
